# Late versus non-late presentation of HIV/AIDS: an economic impact analysis

**DOI:** 10.1186/1758-2652-13-S4-P236

**Published:** 2010-11-08

**Authors:** K Grabmeier-Pfistershammer, A Rieger, T Schroeck, M Schlag

**Affiliations:** 1 DIAID, Medical University Vienna, Währinger Gürtel 18-20, Vienna, Austria; 2 Danube University Krems, Austria, Vienna, Austria;; 3 Gilead Sciences, Vienna, Austria, Vienna, Austria

## Background

In Austria up to 25% of newly diagnosed HIV infected patients present with less than 200 CD4 cell/mm^3^ and/or AIDS (late presenters). Late diagnosis not only adversely affects individual health and survival but may also be associated with higher need for care, thus resulting in higher expenses for the healthcare system.

## Purpose of the study

To assess the marginal costs of late presentation of HIV-infection in Austria.

## Methods

Direct costs incurred during follow-up, demographic and clinical data were retrospectively collected for all late presenters (=cases) and an age- and risk-group matched cohort of controls (>350 CD4 cells/mm^3^ and never AIDS at presentation) presenting at the HIV-unit of the Medical University of Vienna between July 2006 and November 2008. Calculation of costs was based on official standard reimbursement systems for in-patient care, out-patient consultations, diagnostic procedures, and drug prices paid by the social insurance company or the hospital.

## Results

24 cases and 27 controls were followed over a mean of 15 months. Cases and controls were well matched with regards to age, gender, risk group, migrational background, and follow-up time (p=NS). Median overall costs for late presenters incurred during the observation period were nearly 5 times higher for cases than for controls (21.166 vs. 4.329 Euros; p < 0,05). This difference was driven by higher costs for out-patient consultations (p<0,005), in-patient care (p<0,0001), diagnostic procedures (p< 0,02) and ART (p < 0,005). Costs for non-antiretroviral drugs did not differ significantly between the groups.

## Conclusions

Late presentation of HIV-infection is associated with a significant economic burden for the health system during the initial period of care. Higher costs for ART due to the immediate need for treatment account only for part of these costs. Interventions promoting earlier diagnosis of HIV in Austria may therefore prove to be cost-effective.

